# Human papillomavirus 9-valent vaccine for cancer prevention: a systematic review of the available evidence

**DOI:** 10.1017/S0950268817000747

**Published:** 2017-04-27

**Authors:** C. SIGNORELLI, A. ODONE, V. CIORBA, P. CELLA, R. A. AUDISIO, A. LOMBARDI, L. MARIANI, F. S. MENNINI, S. PECORELLI, G. REZZA, G.V. ZUCCOTTI, A. PERACINO

**Affiliations:** 1 Department of Medicine and Surgery, University of Parma, Parma, Italy; 2 School of Medicine, University Vita-Salute San Raffaele, Milan, Italy; 3 Department of Health Services Research and Policy, London School of Hygiene and Tropical Medicine, London, UK; 4 European Institute of Oncology – Medical Office, Milan, Italy; 5 University of Liverpool, Liverpool, UK; 6 Giovanni Lorenzini Medical Science Foundation, Houston, TX, USA; 7 HPV-Unit, Regina Elena National Cancer Institute, Rome, Italy; 8 Centre for Economic and International Studies, University of Rome “Tor Vergata”, Rome, Italy; 9 Institute of Leadership and Management in Health Care, Kingston University, London, UK; 10 School of Medicine, University of Brescia, Brescia, Italy; 11 Department of Infectious Diseases, Istituto Superiore di Sanità, Rome, Italy; 12 Department of Paediatrics, University of Milan, Milan, Italy; 13 Giovanni Lorenzini Medical Science Foundation, Milan, Italy

**Keywords:** Cancer prevention, HPV, human papillomavirus 9-valent vaccine, systematic review

## Abstract

In 2014, the Food and Drug Administration approved a new human papillomavirus 9-valent vaccine (9vHPV), targeting nine HPV types: HPV types 6, 11, 16, and 18, which are also targeted by the quadrivalent HPV vaccine (qHPV), plus five additional high cancer risk HPV types (HPV types 31, 33, 45, 52, and 58). The aim of the current study was to systematically retrieve, qualitatively and quantitatively pool, as well as critically appraise all available evidence on 9vHPV from randomized controlled trials (RCTs). We conducted a systematic review of the literature on 9vHPV efficacy, immunogenicity and safety, as well as a systematic search of registered, completed, and ongoing RCTs. We retrieved and screened 227 records for eligibility. A total of 10 publications reported on RCTs’ results on 9vHPV and were included in the review. Sixteen RCTs on 9vHPV have been registered on RCT registries. There is evidence that 9vHPV generated a response to HPV types 6, 11, 16 and 18 that was non-inferior to qHPV. Vaccine efficacy against five additional HPV type-related diseases was directly assessed on females aged 16–26 years (risk reduction against high-grade cervical, vulvar or vaginal disease = 96·7%, 95% CI 80·9%–99·8%). Bridging efficacy was demonstrated for males and females aged 9–15 years and males aged 16–26 years (the lower bound of the 95% CIs of both the geometric mean titer ratio and difference in seroconversion rates meeting the criteria for non-inferiority for all HPV types). Overall, 9vHPV has been proved to be safe and well tolerated. Other RCTs addressed: 9vHPV co-administration with other vaccines, 9vHPV administration in subjects that previously received qHPV and 9vHPV efficacy in regimens containing fewer than three doses. The inclusion of additional HPV types in 9vHPV offers great potential to expand protection against HPV infection. However, the impact of 9vHPV on reducing the global burden of HPV-related disease will greatly depend on vaccine uptake, coverage, availability, and affordability.

## BACKGROUND

The latest World Health Organization (WHO) global estimates report 14·1 million new cancer cases to occur every year, 8·2 million cancer deaths and 32·6 million people living with cancer; these figures are projected to increase to 15·2 million new cases and 8·9 deaths by 2035 [1]. Cancers attributable to infections – which can be targeted by immunization-based primary prevention interventions – account for 16% of this burden [2]. Cervical cancer is the fourth most common cancer in women, and the seventh overall, with an estimated 528 000 new cases and 266 000 deaths in 2012 [1]. Human papillomavirus (HPV) carcinogenicity has been solidly established for cervical cancer; HPV infection is responsible for virtually all cervical cancers and for a large number of other genitourinary cancers including vulvar, vaginal, penile and anal cancer, as well as for oropharyngeal cancers, resulting in HPV being responsible for a significant proportion of worldwide cancer burden [3–10]. There are more than 100 types of HPV, of which at least 12 have been identified as high-risk oncogenic types and are vaccine targets. HPV types 16 and 18 were classified as carcinogens by the International Agency for Research on Cancer (IARC) in 1995 [11] and are reported to account for approximately 70% of cervical cancers. HPV types 31, 33, 35, 39, 45, 51, 52, 56, 58, and 59 were included in the IARC carcinogens group in 2011 [12] and are reported to account for 30% of cervical cancers [13–17].

The quadrivalent (against HPV types 6, 11, 16, and 18, qHPV) and bivalent (against HPV types 16 and 18, bHPV) vaccines were licensed in the US in 2006 and 2009 and have since been widely introduced in immunization schedules at the global level. In Europe, qHPV is approved for use in males and females from the age of 9 years to protect against precancerous lesions in the cervix, vulva, vagina and anus, cervical and anal cancers and genital warts [18]; bHPV is approved for use in males and females from the age of 9 years to protect against cervix or anus cancers and against precancerous lesions in the genital area [19].

A second-generation HPV 9-valent vaccine (9vHPV) targeting five additional HPV types (against HPV types 6, 11, 16, 18, 31, 33, 45, 52, and 58) was approved in December 2014 by the US Food and Drug Administration (FDA) [20] and granted marketing authorization by the European Commission in June 2015 [21]. In Europe, 9vHPV is approved for use in males and females from the age of 9 years to protect against precancerous lesions and cancers of the cervix, vulva, or vagina and anus, and against genital warts [22].

Currently, 9vHPV is also licensed in Canada, Australia, Chile, and Hong-Kong and, more recently, Ecuador, South Korea, and New Zealand. The US Advisory Committee on Immunization Practices (ACIP) recommended 9vHPV as one of three HPV vaccines that can be used for routine vaccination in February 2015 [23] and, from May 2017, after all lots of qHPV have expired, will be the only HPV vaccine available in the USA [24].

The potential public health impact and cost-effectiveness of 9vHPV has been explored by mathematical models under different efficacy, cost, and vaccine coverage scenarios [25–30]. As clinical trial data is currently accumulating on 9vHPV efficacy, immunogenicity, and safety in different age groups and study populations, as well as on different dose regimens and co-administrations, no systematic assessment has been conducted so far to pool the available evidence on the topic. The aim of the current study is to systematically retrieve, qualitatively and quantitatively pool, and critically appraise all available evidence from randomized controlled trials (RCTs) on 9vHPV.

## METHODS

We conducted a systematic review of the available published evidence on the efficacy, immunogenicity, and safety of 9vHPV, as well as a systematic search of the registered, completed, active, and/or ongoing clinical trials (RCTs) on 9vHPV. The review's methods were defined in advance following the PRISMA (Prepared Items for Systematic Reviews and Meta-Analysis) guidelines [31].

### Search methods for studies’ identification

Published studies were identified by searching the electronic databases Medline, Embase and the Cochrane Library. The database search strategies were built around 9vHPV-related free-text key words. The search strategy was first developed for Medline and then adapted for use in Embase and the Cochrane Library. All three search strategies are available as online supplementary material (Table S1). In addition, further studies were retrieved from reference listing of relevant articles and consultation with experts in the field.

Registered clinical trials were identified searching the clinical trials’ registries and platforms: the WHO ICTRP (International Clinical Trials Registry Platform), the ClinicalTrials.gov registry, the Cochrane Central Register of Controlled Trials and the EU Clinical Trial Register (all registries’ search strategies are available as online supplementary material, Table S1).

### Inclusion criteria and outcomes

All published clinical trials’ on 9vHPV were included in the systematic review of the literature. All other study designs were considered not eligible for inclusion, neither were reviews or opinion papers. We considered the following primary outcomes: all measures of 9vHPV's clinical efficacy, immunogenicity, and safety in all possible age groups and study populations. Studies published in English through August 25, 2016 were included.

In our systematic search of the registered RCTs, we retrieved all RCT protocols through August 25, 2016 reporting 9vHPV to be administered in any of the RCTs’ arms. All age groups, study populations, comparisons, and dose regimens were included.

### Collection and analysis

All identified studies were independently reviewed for eligibility by three authors (P.C., V.C., and A.O.) in a two-step-based process; a first screen was performed based on title and abstract while full texts were retrieved for the second screen. At both stages disagreements by reviewers were resolved by consensus. Data were extracted by two authors (P.C. and V.C.) supervised by a third author (A.O.), using a standardised data extraction spreadsheet. The data extraction spreadsheet was piloted on three randomly selected papers and modified accordingly. Published studies’ data extraction included authors’ name, year of publication, countries of study implementation, study setting, study period, study population, study design, intervention, control, and outcome measures. Data extraction from RCTs’ registered protocols included Trial's title, ClinicalTrials.gov identifier, EudraCT number, sponsor, sponsor protocol number, start date and current status. For each included registered RCT, we retrieved and reported on associated indexed published papers.

We performed a descriptive analysis to report the characteristics of included studies. A synthesis of the studies’ findings was carried out and results summary tables were produced on all considered outcomes. Depending on studies’ heterogeneity, we planned to perform meta-analysis on pre-specified outcomes, including efficacy, immunogenicity, and safety outcomes.

## RESULTS

### Characteristics of included studies

**Fig. 1.**
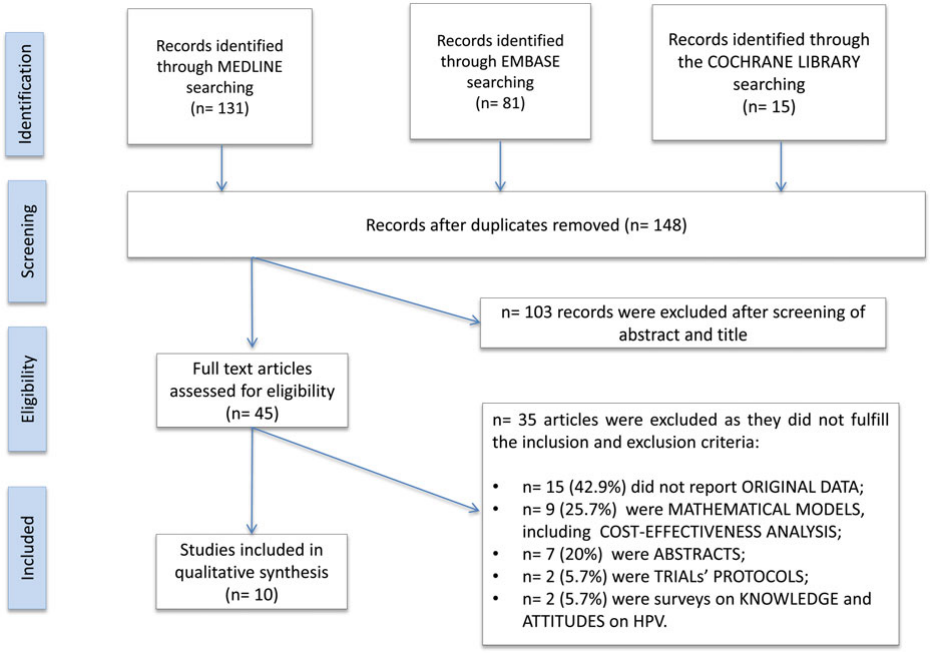
PRISMA flowchart of included published papers.

We identified 227 records by running the pre-defined search strategies on the three selected databases. After removing duplicates, 148 papers were assessed for eligibility by title and abstract. Papers were screened and selected as illustrated in Figure 1. Two papers were published on 9vHPV clinical trials’ protocols, describing the RCTs’ design and rationale but not reporting original data; they were excluded from the review [32, 33]. Ten papers met the inclusion criteria and were included in the review.

Included papers’ characteristics are reported in Table 1.

**Table 1.** Characteristics of included published studies, reporting findings from randomized, controlled trial on 9vHPV

Reference	Trial's NCT number	Study design	Study site	Study period	Study population	Sample size*	Follow up	Intervention	Comparison	Assessed outcomes	Analysis
Joura *et al.* [34]	NCT00543543	Phase III Randomized, double-blind controlled trial	Multicenter 18 countries	September 2007–April 2013	Females, 16–26 years	14215	54 months	9vHPV (3 doses at 0, 2, and 6 months)	qHPV (3 doses at 0, 2, and 6 months)	EFFICACY IMMUNOGENICITY SAFETY	Modified intention-to-treat: efficacy Per-protocol for all assessed outcomes
Luxembourg *et al.* [38]	NCT00543543	Three Phase II Randomized, double-blind controlled trials (Study 1, Study 2, Study 3)	Multicenter Study 1:3 countries Study 2:7 countries Sudy 3:5 countries	Study 1: December 2005–August 2007 Study 2: September 2007–April 2013 Study 3: October 2007–May 2009	Females, 16–26 years	Study 1: 680 Study 2: 1242 Sudy 3: 623	Study 1: 7 months Study 2: 3 months Study 3: 7 months	Study 1: 8vHPV Study 2: 9vHPV (low-, mid-, and high-dose form.) Sudy 3: qHPV + 5vHPV Study 1, 2, and 3: (3 doses at 0, 2, and 6 months)	Study 1, 2, and 3: qHPV (3 doses at 0, 2, and 6 months)	IMMUNOGENICITY SAFETY	Per-protocol for all assessed outcomes
Luxembourg *et al.* [39]	NCT00943722	Phase III Randomized, double-blind controlled trial	Multicenter 17 countries	(not reported)	Females, 9–15 years	1935	7 months	9vHPV (3 doses at 0, 2, and 6 months)	9vHPV (3 doses at 0, 2, and 6 months)	IMMUNOGENICITY (lot consistency study, 3 lots)	Per-protocol
Van Damme *et al.* [42]	NCT00943722	Phase III Randomized, double-blind controlled trial	Multicenter 17 countries	August 2009–April 2013	Females, 16–26 years females and males 9–15 years	3074	7 months	9vHPV (3 doses at 0, 2, and 6 months) in females, 16–26 years	9vHPV (3 doses at 0, 2, and 6 months) in females and males 9–15 years	IMMUNOGENICITY SAFETY (adult–adolescent immunobridging study) ANTIBODY PERSISTANCE	Per-protocol for all assessed outcomes
Schilling *et al.* [40]	NCT00988884	Randomized, double-blind controlled trial	Multicenter 4 countries	October 2009–February 2011	Males and females, 11–15 years	1241	8 months	9vHPV (3 doses at 0, 2, and 6 months) + concomitant MCV4/Tdap (1 dose at 0 month)	9vHPV (3 doses at 0, 2, and 6 months) + non-concomitant MCV4/Tdap (1 dose at 1 month)	IMMUNOGENICITY SAFETY	Per-protocol for all assessed outcomes
Garland *et al.* [36]	NCT01047345	Randomized, double-blind controlled trial	Multicenter 8 countries	February 2010–June 2011	Females, 12–26 years who previously received a qHPV three-dose regimen	924	7 months	9vHPV (3 doses at 0, 2, and 6 months)	Placebo	IMMUNOGENICITY SAFETY	Per-protocol for all assessed outcomes
Kosalaraksa *et al.* [37]	NCT01073293	Randomized, double-blind controlled trial	Multicenter 6 countries	April 2010–June 2011	Males and females, 11–15 years	1054	8 months	9vHPV (3 doses at 0, 2, and 6 months) + concomitant Tdap-IPV (1 dose at 0 month)	9vHPV (3 doses at 0, 2, and 6 months) + non-concomitant Tdap-IPV (1 dose at 1 month)	IMMUNOGENICITY SAFETY	Per-protocol for all assessed outcomes
Vesikari *et al.* [43]	NCT01304498	Phase III Randomized, double-blind controlled trial	Multicenter 6 countries	February 2011–May 2011	Females, 9–15 years	600	7 months	9vHPV (3 doses at 0, 2, and 6 months)	qHPV (3 doses at 0, 2, and 6 months)	IMMUNOGENICITY SAFETY	Per-protocol for all assessed outcomes
Castellsagué *et al.* [35]	NCT01651949	Randomized, double-blind controlled trial	Multicenter 17 countries	October 2012–August 2014	Females, 16–26 years Males 16–26 years	2520	7 months (12 months safety)	9vHPV (3 doses at 0, 2, and 6 months) in heterosexual males 16–26 years and men having sex with men 16–26 years	9vHPV (3 doses at 0, 2, and 6 months) in females, 16–26 years	IMMUNOGENICITY SAFETY (females–males immunobridging study)	Per-protocol for all assessed outcomes
Van Damme *et al.* [41]	NCT02114385	Phase III Randomized, double-blind controlled trial	Multicenter 3 countries	March 2014–April 2015	Males 16–26 years	500	7 months	9vHPV (3 doses at 0, 2, and 6 months)	qHPV (3 doses at 0, 2, and 6 months)	IMMUNOGENICITY SAFETY	Per-protocol for all assessed outcomes

9vHPV, human papillomavirus 9-valent vaccine; 8vHPV, HPV 8-valent vaccine; 5vHPV, HPV 5-valent vaccine; qHPV, HPV quadrivalent vaccine; MCV4, meningococcal A/C/Y/W-135 vaccine; Tdap, tetanus, diphtheria, acellular pertussis vaccine; Tdap-IPV, diphtheria, tetanus, pertussis, and poliomyelitis vaccine; Pap test, Papanicolaou test; Yrs, years; Dose form., dose formulations.

* Subjects who underwent randomization.

Included papers reported findings from eight different clinical trials on 9vHPV's efficacy, immunogenicity, and/or safety. Of them, one paper reported data on 9vHPV clinical efficacy [34], 10 papers reported data on 9vHPV immunogenicity [34–43], and nine papers reported data on 9vHPV safety [34–38, 40–43].

In particular:

- one paper reported the findings of the three Phase II RCTs that were conducted to select the best vaccine formulation to undergo Phase III evaluation [38];
- one paper reported preliminary data on 9vHPV clinical efficacy in females aged 16–26 years, as well as on 9vHPV immunogenicity and safety in this population, compared with qHPV [34];
- one paper reported data on 9vHPV immunogenicity and safety in males aged 16–26 years, compared with qHPV [41];
- one paper reported data on immunogenicity and safety of 9vHPV in 9–15-year-old females, compared with qHPV [43];
- two papers reported on 9vHPV bridging efficacy to 9–15-year-old populations [42], 16–26-year-old heterosexual males and men having sex with men (MSM) [35], respectively;
- one paper reported the findings of the Phase III trial conducted to evaluate vaccines’ lot consistency [39];
- two papers reported data on concomitant administration of 9vHPV with other vaccines, compared with non-concomitant administration [37, 40];
- one paper reported data on 9vHPV immunogenicity and safety in young females previously immunized with qHPV vaccine [36].

The sections below, together with Tables 2–4, report the synthesis of included RCTs’ findings, by outcome. Meta-analysis of RCTs’ estimates could not be performed due to studies’ heterogeneity in terms of: study design, tested interventions, and comparisons, as well as targeted study populations.

**Table 2.** HPV 9-valent vaccine clinical efficacy and bridging efficacy findings of included studies

Subgroup	Efficacy comparison with qHPV vaccine risk reduction, (95% CI)			
	Females, 16–26 years [34]			
	High-grade cervical, vulvar, and vaginal disease	High-grade cervical epithelial neoplasia, adenocarcinoma *in situ*, and cervical cancer		Persistent infection ⩾6 months’ duration
Related to HPV-31, 33, 45, 52, or 58	96·7 (80·9–99·8)	96·3 (79·5–99·8)		96·0 (94·4–97·2)
Related to HPV-6, 11, 16, or 18	66·6 (−203·0 to 98·7)	−0·4 (⩽−999 to 97·4)		26·4 (−4·3 to 47·5)
All participants	0·7 (−15·7 to 14·8)*	−0·3 (−17·3 to 14·3)*		
HPV uninfected on day 1	42·5 (7·9–65·9)*	39·7 (1·8–64·3)*		
Not related to 9 vaccine HPV types	19·7 (−34·5 to 52·5)*	14·3 (−49·1 to 49·1)*		
Related to 9 vaccine HPV types	100 (70·4–100)*	100 (70·3–100)*		
HPV infected on day 1	−4·8 (−23·3 to 10·8)*	−5·3 (−24·1 to 10·8)*		
Not related to 9 vaccine HPV types	−2·0 (−30·0 to 19·9)*	1·8 (−26·0 to 23·5)*		
Related to 9 vaccine HPV types	−6·8 (−33·2 to 14·3)*	−11·3 (−39·6 to 11·0)*		

	Bridging efficacy comparison with 9vHPV administered to females, 16–26 years			
	Month 7 GMT ratio (95% CI)			
	Females, 9–15 years [42]	Males, 9–15 years [42]	Heterosexual males, 16–26 years [35]	MSM, 16–26 years [35]
Anti-HPV-6	1·90 (1·70–2·14)	2·31 (2·07–2·59)	1·11 (1·02–1·21)	0·81 (0·70–0·93)
Anti-HPV-11	1·83 (1·63–2·06)	2·10 (1·88–2·36)	1·09 (1·00–1·19)	0·77 (0·67–0·89)
Anti-HPV-16	1·98 (1·77–2·22)	2·45 (2·19–2·74)	1·20 (1·10–1·30)	0·82 (0·72–0·94)
Anti-HPV-18	2·44 (2·13–2·80)	3·20 (2·80–3·65)	1·19 (1·08–1·31)	0·89 (0·77–1·04)
Anti-HPV-31	2·51 (2·21–2·85)	2·95 (2·60–3·34)	1·24 (1·13–1·37)	0·74 (0·64–0·86)
Anti-HPV-33	2·10 (1·87–2·36)	2·57 (2·29–2·88)	1·19 (1·10–1·30)	0·78 (0·69–0·89)
Anti-HPV-45	2·62 (2·27–3·03)	3·33 (2·89–3·84)	1·27 (1·14–1·41)	0·85 (0·72–0·99)
Anti-HPV-52	2·22 (1·97–2·51)	2·47 (2·19–2·79)	1·15 (1·05–1·26)	0·70 (0·61–0·80)
Anti-HPV-58	2·18 (1·93–2·45)	2·66 (2·37–2·98)	1·25 (1·14–1·36)	0·78 (0·68–0·89)

	Month 7 seroconversion % difference (95% CI)			
	Females, 9–15 years [42]	Males, 9–15 years [42]	Heterosexual males, 16–26 years [35]	
Anti-HPV-6	0·1 (−0·8 to 1·5)	0·1 (−0·7 to 1·5)	0·1 (−0·7 to 0·9)	
Anti-HPV-11	0 (−0·7 to 1·2)	0 (−0·7 to 1·2)	0·1 (−0·3 to 0·8)	
Anti-HPV-16	0 (−0·7 to 1·2)	0 (−0·7 to 1·2)	0·1 (−0·3 to 0·7)	
Anti-HPV-18	0·1 (−0·8 to 1·5)	0·3 (−0·4 to 1·6)	0·1 (−0·4 to 0·8)	
Anti-HPV-31	0·3 (−0·4 to 1·7)	0·3 (−0·4 to 1·7)	0 (−0·4 to 0·5)	
Anti-HPV-33	0·3 (−0·4 to 1·6)	0·3 (−0·4 to 1·6)	0·1 (−0·3 to 0·7)	
Anti-HPV-45	0·4 (−0·6 to 1·8)	0·5 (−0·1 to 2·0)	0·2 (−0·4 to 1·0)	
Anti-HPV-52	0·3 (−0·4 to 1·7)	0·3 (−0·4 to 1·7)	0·2 (−0·2 to 0·9)	
Anti-HPV-58	0 (−0·7 to 1·2)	0 (−0·7 to 1·2)	0·2 (−0·2 to 0·9)	

MSM, men-having-sex with men; 9vHPV, human papillomavirus 9-valent vaccine; qHPV, HPV quadrivalent vaccine; GMT, geometric mean titer.

* Intention-to-treat populations (all other estimates are on per protocol populations).

**Table 3.** HPV 9-valent vaccine immunogenicity findings of included studies

Study population	Analysis	HPV type	Immunogenicity comparison with qHPV vaccine	
			Month 7 GMT ratio	Month 7 seroconversion % difference
Females, 16–26 years [34]*	Per-protocol	Anti-HPV-6	1·02 (0·99–1·06)	0·0 (−0·3 to 0·2)
		Anti-HPV-11	0·80 (0·77–0·83)	0·0 (−0·1 to 0·2)
		Anti-HPV-16	0·99 (0·96–1·03)	0·0 (−0·1 to 0·2)
		Anti-HPV-18	1·19 (1·14–1·23)	0·1 (−0·1 to 0·4)
Females, 9–15 years [43]		Anti-HPV-6	1·07 (0·93–1·23)	
		Anti-HPV-11	0·93 (0·80–1·08)	
		Anti-HPV-16	0·97 (0·85–1·11)*	
		Anti-HPV-18	1·08 (0·91–1·29)*	
Males, 16–26 years [41]*	Per-protocol	Anti-HPV-6	1·23 (1·04–1·45)	−0·5 (−0·6 to 0·2)
		Anti-HPV-11	0·89 (0·76–1·04)	0·0 (0–0)
		Anti-HPV-16	1·04 (0·89–1·21)	0·0 (−0·1 to 0)
		Anti-HPV-18	1·12 (0·91–1·37)	0·0 (−0·1 to 0)

qHPV, human papillomavirus quadrivalent vaccine; GMT, geometric mean titer.

* The *P* value for non-inferiority was <0·001 for all comparisons HPV 9-valent vaccine *vs*. qHPV vaccine.

**Table 4.** The 9vHPV safety findings of included studies; adverse events (AEs, %)

Event	Comparison: 9vHPV *vs*. qHPV					
	Females, 16–26 years [34]		Females, 9–15 years [43]*		Males, 16–26 years [41]*	
	9vHPV	qHPV	9vHPV	qHPV	9vHPV	qHPV
One or more AEs	93·9%	90·7%	96·0%	93·7%	82·3%	81·9%
Injection-site events	90·7%	84·9%	91·6%	88·3%	79·0%	72·2%
Systemic events	55·8%	54·9%	47·5%	52%	40·7%	40·3%
Serious events	3·3%	2·6%	0·3%	0·7%	0·0%	0·0%
Discontinuation due to AEs	0·1%	0·1%	0·3%	0·3%	0·0%	0·0%

	Comparison: 9vHPV in Females, 16–26 years *vs*. other groups					
	Females, 16–26 years [42]	Females, 9–15 years [42]	Males, 9–15 years [42]	Females, 16–26 years [35]	Males, 16–26 years [35]	
One or more AEs	90·01%	86·6%	81·0%	89·4%	76·2%	
Injection-site events	85·4%	81·9%	72·8%	84·1%	67·6%	
Systemic events	57·1	45·0%	41·8%	37·1%	48·8%	
Serious events	3·2%	0·9%	1·7%	2·4%	1·6%	
Discontinuation due to AEs	0·0%	0·0%	0·2%	0·3%	0·1%	

	Comparison: 9vHPV *vs*. placebo in females, 12–26 years, previously vaccinated with qHPV [36]					
	9vHPV	Placebo				
One or more AEs	95·9%	75·1%				
Injection-site events	91·9%	43·9%				
Systemic events	59·7%	55·7%				
Serious events	0·5%	1·0%				
Discontinuation due to AEs	0·5%	0·0%				

	Comparison: concomitant *vs*. non-concomitant 9vHPV administration with other vaccines					
	Post-vaccination 1 [40]		Post-vaccination 2 [40]		Post-vaccination 3 [40]	
	Concomitant	Non-concomitant	Concomitant	Non-concomitant	Concomitant	Non-concomitant
One or more AEs	85·3%	85·1%	51·9%	50·4%	55·4%	52·8%
Injection-site events	80·9%	80·4%	46·7%	46·5%	52·1%	48·4%
Systemic events	43·1%	42·4%	16·1%	15·0%	14·8%	16·2%
Serious events	0·2%	0·2%	0·2%	0·0%	0·0%	0·0%
Discontinuation due to AEs	0·0%	0·2%	0·2%	0·0%	0·0%	0·0%
	Post-vaccination 1 [37]		Post-vaccination 2 [37]		Post-vaccination 3 [37]	
	Concomitant	Non-concomitant	Concomitant	Non-concomitant	Concomitant	Non-concomitant
One or more AEs	95·2%	93·0%	66·3%	66·2%	73·7%	71·9%
Injection-site events	93·9%	90·1%	60·7%	60·2%	68·3%	66·1%
Systemic events	48·6%	48·6%%	19·2%	18·0%	21·5%	19·8%
Serious events	0·6%	0·0%	0·0%	0·0%	0·2%	0·4%
Discontinuation due to AEs	0·0%	0·0%	0·0%	0·0%	0·0%	0·0%

9vHPV, human papillomavirus 9-valent vaccine; qHPV, HPV quadrivalent vaccine.

* The difference in AE incidence between vaccines was statistically significant only for injection-site swelling (*P* = 0·003).

### Dose formulation data

Data from the Phase II dose formulation study were published in June 2015 [38]. The paper reports the findings of three RCTs conducted to compare immunogenicity and safety of seven vaccine candidates with licensed qHPV vaccine. The vaccine candidates were: three dose formulations (low-, mid-, and high-dose formulations) of an 8-valent HPV6/11/16/18/31/45/52/58 vaccine (Study 1), three dose formulations of a 9-valent HPV6/11/16/18/31/33/45/52/58 vaccine (Study 2), and qHPV vaccine concomitantly administered with a 5-valent HPV31/33/45/52/58 (Study 3). All vaccines were administered on a 3-dose schedule at months 0, 2, and 6. Six-hundreds and eighty, 1242 and 623 girls aged 16–26 years were, respectively, randomized in the three studies. Within each study, the primary immunogenicity objective was to demonstrate that month 7 anti four original vaccine HPV types (anti-HPV6/11/16/18) geometric mean titers (GMTs) were non-inferior in subjects who received experimental vaccines compared with subjects in the control group. Non-inferiority was defined as the lower bound of the two-sided 95% CI of the GMT ratio (experimental arm/control arm) being >0·5 for each of anti-HPV types 6/11/16/18. Among all vaccine candidates, the 9vHPV mid-dose formulation containing 30/40/60/40/20/20/20/20/20 mg of HPV6/11/16/18/31/33/45/52/58 virus such as particles, and 500 mg of amorphous aluminum hydroxyl-phosphate sulfate adjuvant was selected for all subsequent Phase III efficacy, immunogenicity, and safety evaluations.

Successful 9vHPV vaccine candidate was reported to: (i) provide non-inferior antibody responses compared with qHPV vaccine with respect to the four HPV types covered by both vaccines (the lower bound 95% CI GMT ratio exceeded 0·5 for all HPV types), (ii) be strongly immunogenic against five additional oncogenic HPV types (>95% subjects seroconverted at month 3), and (iii) be generally well tolerated. Injection-site adverse events (AEs) were slightly higher in the selected 9vHPV vaccine as compared with qHPV (92·4% *vs*. 90·3%).

### Efficacy

The 9vHPV efficacy findings of included studies are summarized in Table 2. One paper has been published on 9vHPV clinical efficacy so far [34]. It reports preliminary data of a Phase III multinational multicenter efficacy study conducted on 14 215 women aged 16–26 years comparing 9vHPV to qHPV. The primary efficacy outcome was the combined incidence of several conditions related to 9vHPV additional HPV types (HPV-31, 33, 45, 52, 58) – with follow-up over a 54-month period. Analysis in the modified intention-to-treat population (which included subjects both not HPV-infected and HPV-infected at the time of vaccination, who received at least one dose of vaccine and for whom there was at least one measurement of efficacy) showed high-grade cervical, vulva, and vaginal disease incidence to be the same in the 9vHPV and qHPV arms, irrespective of HPV testing results (14·0 per 1000 persons-years, risk reduction = 0·7, 95% CI −15·7 to 14·8), while when restricting the analysis to not HPV-infected participants at the time of vaccination, 9vHPV risk reduction was 42·5%; (95% CI 7·9–65·9), this reaching 100% (95% CI 70·4–100) when only considering disease related to 9vHPV HPV types. Analysis conducted on the per-protocol population (subjects who received all three doses of vaccine within 1 year, who were seronegative on day 1 and PCR-negative from day 1 through month 7 and had no protocol violations) reported 9vHPv vaccine efficacy to be 96% or above for all considered clinical outcomes related to HPV types 31, 33, 45, 52, or 58 as well as persistent infection (⩾6 months’ duration, Table 2) [34].

Two published immunobridging efficacy studies inferred 9vHPV efficacy in males and females aged 9–15 years [42], in heterosexual males and in MSM aged 16–26 years [35], comparing immunogenicity data between individuals in the intervention arms and 16–26-year-old female controls. By definition, bridging studies generate immunogenicity data to support the extrapolation of data on efficacy or safety obtained under specific circumstances of use (in our case 9vHPV efficacy in 16–26-year-old females) to other situations (e.g. different age groups or different populations) [44]. Both studies reported month 7 GMTs in the intervention arms to be non-inferior (lower bound of the two-sided 95% CI of the GMT ratio >0·67) to the control arm for all 9vHPV vaccine types, as well as seroconversion non-inferiority in more than 99% of study participants (defined as the lower bound of the two-sided 95% CI for the differences in seroconversion rates being >−5 percentage points for each 9vHPV HPV type) (Table 2). Data on 9vHPV immunogenicity MSM aged 16–26 years showed lower antibody response compared with heterosexual males for all tested HPV types (GMT ratios MSM/women for all 9vHPV vaccine types are reported in Table 2) [35].

### Immunogenicity

All included articles assessed 9vHPV immunogenicity. The majority of studies adopted a non-inferiority design. Immunogenicity outcomes included intervention–control comparisons of month 7 GMTs for anti-HPV types and proportion of studies’ participants with seroconversion to the 9vHPV vaccine types at 4 weeks after the administration of the third vaccine dose (month 7).

The immunogenicity analyses findings of the dose-formulation Phase II study has been described in the previous section [38] and reference was previously made to the published lot-consistency study – conducted to meet regulatory requirements – that reported three different lots of 9vHPV to elicit equivalent antibody response and seroconversion rates [39]. In addition, data are available on: (i) 9vHPV *vs*. qHPV immunogenicity [34, 41, 43]; (ii) 9vHPV immunogenicity in females aged 16–26-years old *vs*. other age and gender populations to infer bridging efficacy [35, 42]; (iii) 9vHPV immunogenicity when co-administrated with other vaccines (including meningococcal, tetanus, diphtheria, pertussis, polio vaccines) [37, 40]; and (iv) 9vHPV immunogenicity in subjects previously vaccinated with qHPV [36].

Three studies reported immunogenicity data comparing 9vHPV to qHPV in females aged 16–26 years [34], in females aged 9–15 years [43] and in males aged 16–26 years [41]. These are summarized in Table 3. They showed: (i) 9vHPV non-inferiority as compared with qHPV with regard to HPV types 6, 11, 16, and 18 in females and males aged 16–26 years (GMT ratios ranging from 0·80 and 1·19 and from 0·89 and 1·23, respectively, Table 3) [34, 41]; (ii) 9vHPV non-inferiority as compared with qHPV with regard to HPV types 16 and 18 in females aged 9–15 years [43]; and (iii) virtually all studies’ participants seroconversion for HPV 31/33/45/52/58 at month 7 (in females aged 9–15 years and 16–26 years and in males aged 16–26 years [34, 41, 43]; Table 3).

Two studies have been published on concomitant administration of 9vHPV with other vaccines, such as meningococcal (MCV4), tetanus, diphtheria, pertussis (Tdap) and polio vaccines, reporting non-inferior immune response in subjects receiving 9vHPV concomitantly with other vaccines as compared with non-concomitant administration [37, 40]. In particular, authors reported concomitant administration of 9vHPV together with MCV4 and Tdap to elicit non-inferior GMTs and seroconversion rates for all 9vHPV antigens as well as non-inferior immune response and seroconversion to MCV4 and Tdap vaccines’ components as compared with non-concomitant vaccines’ administration in subjects aged 11–15-years old [40]. Similarly, findings from another RCT showed all 9vHPV HPV types 7 month GMTs and seroconversion non-inferiority in the concomitant group compared with the non-concomitant group. Non-inferiority of immune response was established for diphtheria, tetanus, all pertussis, and polio antigens for both groups [37].

One study has been published reporting findings from an RCT that assessed 9vHPV immunogenicity *vs*. placebo in females aged 12–26, previously vaccinated with qHPV [36]. Seroconversion at month 7 was reported to be >98% for all 9vHPV HPV types, with marked elevations in GMTs. Data from cross-study analysis showed anti-HPV 31/33/45/52/58 GMTs to be lower than in study subjects administered 9vHPV vaccine who had not previously received qHPV vaccine [36].

### Safety

Nine papers reported data on 9vHPV vaccine safety; of them, one is the Phase II RCTs conducted to select the right 9vHPV formulation [38], three made comparisons between 9vHPV and qHPV vaccine [34, 41, 43], two made comparisons between 9vHPV administered in different age and gender populations (immunobridging studies) [35, 42], two assessed 9vHPV safety comparing concomitant and non-concomitant administration with other vaccines [37, 40] and one assessed 9vHPV safety against placebo in girls previously vaccinated with qHPV vaccine [36]. In five studies, participants recorded injection-site events (AE, within 5 days after vaccination) and systemic events (within 15 days after vaccination) on VRCs (vaccination report cards) [34, 35, 38, 41, 42]. Injection-site AEs were rated by study subjects to be mild/moderate/severe. AEs causality was assessed by investigators and classified as possibly, probably, or definitely vaccine related. Serious AEs were pre-defined as any AE resulting in deaths or in which discontinuation due to AEs was reported. AEs were summarized as frequencies and percentages by study arm and AE type. The 9vHPV safety findings are reported in Table 4. Overall, 9vHPV vaccine recipients were more likely than qHPV vaccine recipients to have AEs related to the injection site (90·7% *vs*. 84·9% in females aged 16–26) [34], while systemic AEs distribution was similar between intervention and control groups (55·8% *vs*. 54·9% in female aged 16–26) [34]. Discontinuation rates because of vaccine-related AE were rare and all but one study [34] reported no vaccine-related serious AEs [34, 38, 41, 43]. In immunobridging studies injection-site or systemic AEs were lower in heterosexual men and MSM aged 16–26 and in males and females aged 9–15 years as compared with controls (females aged 16–26 years) [35, 42]. In studies assessing concomitant vaccines administration, injection-site AEs of swelling after 9vHPV and Tdap-IPV were more frequent in concomitant administration arms as compared with non-concomitant ones (after 9vHPV: 14·4% *vs*. 9·4% [40], after Tdap-IPV 21·7% *vs*. 31·3% [37]); the risk difference between the groups being statistically significant. No other statistically significant differences were reported in terms of AEs after all vaccines’ administration between study arms [37, 40]. In both studies, few subjects discontinued because of an AE and no deaths were reported [37, 40]. In the placebo-controlled trial conducted in females who previously received qHPV, injection-site AEs were more frequent in the intervention arm (91·1% *vs*. 43·9) and increased with subsequent 9vHPV vaccine doses, the incidence of serious vaccine-related AEs and discontinuation was reported to be low and no subjects died during the study [36].

### Ongoing clinical studies

**Fig. 2.**
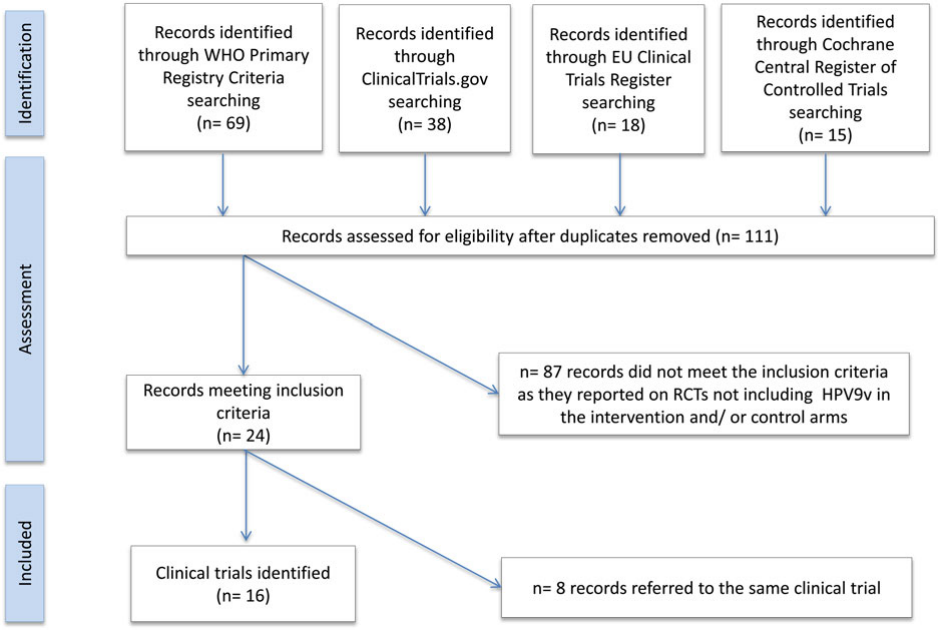
PRISMA flowchart of included registered trials (update August 25, 2016).

One hundred and forty records were initially retrieved searching the selected clinical trials’ registries and platforms (Fig. 2). After removing duplicates, 87 RCTs were excluded as they did not include 9vHPV administration in the intervention or control study arms. Twenty-four relevant clinical trial protocols met the inclusion criteria, referring to 16 clinical trials conducted on the 9vHPV vaccine (some RCTs were registered more than once in different RCT registries; Table 5).

**Table 5.** Existing randomized controlled trials on human papillomavirus 9-valent vaccine

Title	Clinical trials.gov identifier	EudraCT number	Sponsor & sponsor protocol number	Start date	Status	Published results
A Randomized, International, Double-Blinded (With In-House Blinding), Controlled With GARDASIL, Dose-Ranging, Tolerability, Immunogenicity, and Efficacy Study of a Multivalent Human Papillomavirus (HPV) L1 Virus-Like Particle (VLP) Vaccine Administered to 16- to 26- Year-Old Women	NCT00543543	EUCTR2007-003528-39-DK/SE/DE/AT	Merck Sharp & Dohme Corp. V503-001	September 2007	Completed*	Yes [34, 38]
A Phase III Clinical Trial to Study the Immunogenicity, Tolerability, and Manufacturing Consistency of V503 (A Multivalent Human Papillomavirus [HPV] L1 Virus-Like Particle [VLP] Vaccine) in Preadolescents and Adolescents (9 to 15 Year Olds) With a Comparison to Young Women (16 to 26 Year Olds)	NCT00943722	EUCTR2009-011617-25-FI/BE/AT/SE/ES	Merck Sharp & Dohme Corp. V503-002	August 2009	Active, not recruiting*	Yes [39, 42]
A Phase III Clinical Trial to Study the Tolerability and Immunogenicity of 9vHPV (V503), a Multivalent Human Papillomavirus (HPV) L1 Virus-Like Particle (VLP) Vaccine, in 16- to 26-Year-Old Men and 16- to 26-Year-Old Women	NCT01651949	EUCTR2012-002758-22-DE/ES/FI/SE/DK	Merck Sharp & Dohme Corp. V503-003	October 2012	Completed*	Yes [35]
An Open-Label Phase III Clinical Trial to Study the Immunogenicity and Tolerability of GARDASIL®9 (A Multivalent Human Papillomavirus [HPV] L1 Virus-Like Particle [VLP] Vaccine) in Adult Women (27- to 45-Year-Olds) Compared to Young Adult Women (16-to-26 Year-Olds)	–	EUCTR2015-005093-38-DE/AT/ES/BE	Sanofi Pasteur MSD GDS02C/V503-004	July 2016	Ongoing†	No
A Phase III Open-Label Clinical Trial to Study the Immunogenicity and Tolerability of V503 (A Multivalent Human Papillomavirus [HPV] L1 Virus-Like Particle [VLP] Vaccine) Given Concomitantly With Menactra^™^ and Adacel^™^ in Preadolescents and Adolescents (11 to 15 Year Olds)	NCT00988884	–	Merck Sharp & Dohme Corp. V503-005	October 2009	Completed*	Yes [40]
A Phase III, Randomized, International, Placebo-Controlled, Double-Blind Clinical Trial to Study the Tolerability and Immunogenicity of V503, a Multivalent Human Papillomavirus (HPV) L1 Virus-Like Particle (VLP) Vaccine, Given to Females 12–26 Years of Age Who Have Previously Received GARDASIL^™^	NCT01047345	EUCTR2009-015500-26-SE/DK	Merck Sharp & Dohme Corp. V503-006	February 2010	Completed*	Yes [36]
A Phase III Open-Label Clinical Trial to Study the Immunogenicity and Tolerability of V503, a Multivalent Human Papillomavirus (HPV) L1 Virus-Like Particle (VLP) Vaccine, Given Concomitantly With REPEVAX^™^ in Preadolescents and Adolescents (11 to 15 Year Olds)	NCT01073293	EUCTR2009-016218-26-FI/DE/BE/AT/DK	Merck Sharp & Dohme Corp. V503-007	April 2010	Completed*	Yes [37]
A Phase III Open-label, Safety, Tolerability and Immunogenicity Study of a 9-Valent Human Papillomavirus (HPV) L1 Virus-Like Particle (VLP) Vaccine Administered to 9- to 15-Year-Old Japanese Preadolescent and Adolescent Girls	NCT01254643	–	Merck Sharp & Dohme Corp. V503-008	January 2011	Completed*	No
A Randomized, Double-Blinded, Controlled With GARDASIL (Human Papillomavirus Vaccine [Types 6, 11, 16, 18] (Recombinant, Adsorbed)), Phase III Clinical Trial to Study the Immunogenicity and Tolerability of V503 (9-Valent Human Papillomavirus (HPV) Vaccine) in Preadolescent and Adolescent Girls (9- to 15-year-old)	NCT01304498	EUCTR2010-023393-39-FI/BE /SE/ES/DK/IT	Sanofi Pasteur MSD V503-009 GDS01C	February 2011	Completed*	Yes [43]
A Phase III Clinical Trial to Study the Tolerability and Immunogenicity of a 2-dose Regimen of V503, a Multivalent Human Papillomavirus (HPV) L1 Virus-Like Particle (VLP) Vaccine, Administered in Preadolescents and Adolescents (9 to 14 Year Olds) With a Comparison to Young Women (16 to 26 Year Olds)	NCT01984697	EUCTR2013-001314-15-CZ/NO/DK/ES	Merck Sharp & Dohme Corp. V503-010	December 2013	Active, not recruiting*	No
A Randomized, Double-Blinded, Controlled With GARDASIL (Human Papillomavi–us Vaccine [HPV] [Types 6, 11, 16, 18] (Recombinant, Adsorbed)), Phase 3 Clinical Trial to Study the Immunogenicity and Tolerability of V503 (9-Valent Human Papillomavirus L1 Virus-Like Particle [VLP] Vaccine) in 16- to 26-year-old Men	NCT02114385	EUCTR2013-003399-10-DE/BE/NL	Sanofi Pasteur MSD V503-020	March 2014	Completed*	Yes [41]
A Registry-Based Extension of Protocol V503-001 in Countries With Centralized Cervical Cancer Screening Infrastructures to Evaluate the Long-Term Effectiveness, Immunogenicity, and Safety of Multivalent Human Papillomavirus (HPV) L1 Virus- Like Particle (VLP) Vaccine as Administered to 16- to 26- Year- Old Women	NCT02653118		Merck Sharp & Dohme Corp. V503-021	January 2016	Active, not recruiting*	No
Immunogenicity and Safety of Gardasil-9 and Cervarix When Administered to 9–10-year-old Subjects According to 0–6 Month Schedule	NCT02567955	–	Laval University HPV 2355	September 2015	Recruiting*	No
A Prospective, Single-Arm, Open-Label, Non-randomized, Phase IIA Trial of a Nonavalent Prophylactic HPV Vaccine to Assess Immunogenicity of a Prime and Deferred-Booster Dosing Schedule Among 9–11 Year-Old Girls	NCT02568566		National Cancer Institute (NCI) NCI-2015-01645	March 2016	Recruiting*	No
Efficacy of nonavalent vaccine against human papilloma virus (HPV) in HIV infected sexually active men who have sex with men (MSM)	–	EUCTR2015-004524-65-DK	Department of Infectious Diseases Inf.Q002	November 2015	Ongoing†	No
A Dose Reduction Immunobridging and Safety Study of Two HPV Vaccines in Tanzanian Girls	NCT02834637		London School of Hygiene and Tropical Medicine MITU-002	August 2016	Not yet recruiting	No

* As reported by ClinicalTrials.gov.

† As reported by European Union Clinical Trials Register.

As of August 25, 2016, 16 Phase II or Phase III multicenter multinational RCTs have been conducted on 9vHPV, the first of which was started in February 2007 [45] while the last was started in August 2016 [46]. Thirteen (81·2%) RCTs are directly sponsored by pharmaceutical companies, two are sponsored by universities [46, 47], one by the US National Cancer Institute [48] and one by the Danish Department of Infectious Diseases [49].

Of the 16 identified RCTs, eight (50%) have been completed, three (18·8%) are active but not recruiting [50–52], four (25%) are ongoing or recruiting [47–49, 53], and one (6·3%) is not recruiting participants yet [46].

For eight RCTs (50%) results or preliminary results have been published in scientific peer-reviewed journals (Tables 1–4, results described in previous sections); one completed RCTs does not have published studies [54].

Of the active RCTs, three are assessing immunogenicity and tolerability of less than three-dose 9vHPV vaccine schedules [46, 47, 51]. The first one – started in December 2013 and no longer anymore – is a 37-month safety and immunogenicity study conducted with the aim of assessing whether investigational two-dose regimens (0, 6 months and 0, 12 months) administered in males and females aged 9–14 years elicit non-inferior immunogenicity and safety profiles as compared with three-dose regimens administered to females aged 16–26 [51]. The second one – started in September 2015, and still recruiting – aims to assess immunogenicity of 9vHPV and bHPV vaccines administered to males and females aged 9–10 years, according to a 0–6 month schedule to infer possible interchangeable use of the two vaccines [47]. The third one – about to start recruiting females aged 9–14 years in Tanzania – will compare immunogenicity between one-dose and two-dose 9vHPV and bHPV vaccines and three-dose regimens of both vaccines [46].

One active RCT is a long-term follow-up study of the first 9vHPV vaccine RCT (NCT00543543), currently being conducted in countries with centralized cervical cancer screening infrastructures (Denmark, Norway, and Sweden) that aims to evaluate the long-term effectiveness, immunogenicity, and safety of 9vHPV vaccine in females aged 16–26 years [52].

Within ongoing European RCTs, one efficacy immunobridging study started in July 2016 and currently recruiting subjects in four European countries aims at assessing immunogenicity and tolerability of the 9vHPV vaccine in females aged 27–45 years compared with females aged 16–26 years [53]. Another RCT – currently recruiting subjects in Denmark – aims at inferring 9vHPV vaccine efficacy in HIV-infected sexually active MSM, measuring changes in the prevalence of HPV types in either anus, oral cavity, or penis as well as well as antibody response [49].

One Phase IIA RCT started in March 2016 aims at assessing immunogenicity of a prime and deferred-booster dosing 9vHPV vaccine schedule among females aged 9–11 years [48]. Primary and secondary objectives are to determine persistence and stability of the immune response to HPV types 16/18 and HPV types 6/11/31/33/45/52/58, respectively, between 6, 12, 18, and 24 months after the prime dose and prior to the administration of the second dose [48].

## DISCUSSION

We systematically retrieved and comprehensively summarized all the available published evidence on 9vHPV efficacy, immunogenicity, and safety derived from RCTs, as well as systematically presented an update of the ongoing research on the topic.

To date, 10 papers have been published reporting results or preliminary results from RCTs on 9vHPV, of which nine papers reported results from clinical studies included in 9vHPV clinical development program. Overall, there are 16 registered RCTs on 9vHPV, of which eight are currently active or ongoing.

The 9vHPV clinical efficacy has been directly assessed on females aged 16–26 years in one study that reported 96·7% 9vHPV vaccine efficacy against composite high-grade cervical, vulvar, or vaginal disease related to HPV types 31, 33, 45, 52, and 58 [34]. Efficacy against HPV types 6, 11, 16, and 18 was inferred through a non-inferiority approach that demonstrated 9vHPV non-inferior immunogenicity compared with qHPV. Non-inferiority study design represents the primary tool to demonstrate that a new or reformulated vaccine, or a new regimen of already licensed products, is equivalent to the existing vaccine or current vaccine schedule [55, 56].

Although it is well known that HPV vaccine efficacy is highest in HPV infection naïve populations and that young age groups are therefore the target group for prophylactic HPV vaccination, 9vHPV clinical efficacy is not directly tested on these populations due to low exposure to HPV but, instead, inferred extending older subjects efficacy data to younger populations (immunobridging efficacy). In fact, adult-to-adolescent, as well as female-to-male published immunobridging studies have allowed inference of 9vHPV efficacy in males and females aged 9–15 years and in males aged 16–26 years. Overall, 9vHPV has been proven to be safe and well tolerated in both females and males in different age groups, with AE profiles similar to that of the qHPV vaccine.

Other RCTs, including currently ongoing ones, address other aspects relevant to the 9vHPV vaccine, including: vaccine co-administration with other vaccines; administration in subjects that previously received qHPV; and 9vHPV efficacy in less than three-dose regimens.

Evidence from completed RCTs has been used to support marketing authorization applications and to inform National Immunization Technical Advisory Groups (NITAGs). After the FDA licensed 9vHPV for use in females aged 9–26 years and in males aged 9–15 years in 2014, it extended its indication to include use in males aged 16–26 years in December 2015. In February 2015, ACIP recommended 9vHPV as one of three HPV vaccines to be used for routine vaccination at age 11 or 12 years as well as for females aged 13–26 and males aged 13–21 years not previously vaccinated. Vaccination is also recommended through age 26 years for MSM and for immunocompromised subjects [23]. Medical and scientific associations are progressively including the 9vHPV immunization in their recommendations [57–60].

As scientific evidence is accumulating on the 9vHPV vaccine from clinical trials and from other study designs, and as immunization recommendations are built and constantly updated on the basis of their findings, several crucial aspects are to be taken into consideration when trying to forecast the public health impact of universal 9vHPV immunization – to begin with, the broadened protection associated with the five additional HPV types contained in the 9vHPV vaccine. Although some evidence is available on bHPV and qHPV vaccine cross-protection [61], there is no doubt that extending direct protection to five additional HPV types offers great potential. In fact, in the USA, 10% of invasive HPV-associated cancers (14% for females; 4% for males), 15% of cervical cancers, and 25% of ⩾CIN2 are attributable to the five additional types contained in the 9vHPV [62–64]. Although accurate global estimates are missing, a large amount of data is available on HPV genotype-specific prevalence of cervical cancers and other HPV-related cancers in different settings and study populations [16, 65–70]. This has allowed estimation that 9vHPV vaccine use might expand overall protection against cervical cancer to over 90% [70, 71], and – in general – significantly reduce the burden of HPV-related disease [29, 72–75].

For broader roll-out of the 9vHPV vaccine a number of other clinical and organizational aspects are to be considered and are currently being assessed by ongoing research. For instance, long-term follow-up data on 9vHPV efficacy are not yet available. The longest follow-up trial data for bHPV and qHPV vaccines showed them to protect against infection for at least 5 [76] and 9·4 [77] years, respectively. Currently, the longest available follow-up data on 9vHPV reports >90% of vaccinated males and females aged 9–15 to remain seropositive through 2·5 years after third vaccination [42]. In the future, an extension of the first 9vHPV efficacy trial, currently ongoing in three Scandinavian countries with an efficient centralized cancer screening program with the aim of monitoring long-term safety, effectiveness, and immunogenicity of 9vHPV, will allow inference of information on duration of effect as well as to track viral-type replacement [52]. Furthermore, the National Cancer Institute is currently running an RCT which will provide evidence on immunogenicity of a prime and deferred-booster dosing schedule among young girls [48].

The number of doses contained in a 9vHPV regimen is another relevant aspect on which research is currently ongoing and whose findings are likely to impact immunization effectiveness. In fact, there is a general interest in simplified HPV vaccine schedules, which reduce required resources, facilitate immunization programs’ implementation and might increase vaccine acceptability and uptake. In Europe, as well as in other countries, two-dose bHPV and qHPV vaccine schedules have been approved for subjects aged 9–14 and 9–13 years, respectively, based on non-inferiority immunogenicity data. Since 2014, WHO has recommended two-dose regimens for subjects younger than 15. With regards to 9vHPV, research on less than three-dose regimens’ efficacy is ongoing and promising preliminary results from one active RCT were presented to ACIP in February 2016 [51, 78]. The study's findings report two-dose regimens in males and females aged 9–14 to elicit no inferior immunogenicity and a safety profile comparable with the standard three-dose regimen in older females [51]. Other dose reduction immunobridging and safety studies will evaluate reduced-dose regimes in low-income countries where the benefits of lower priced and more accessible immunization programmes would be highly beneficial in reducing the burden of HPV-related disease [46].

Another issue to consider is how to manage the transition from qHPV or bHPV vaccines to 9vHPV. The Centers for Disease Control and Prevention has issued a guidance document on the topic [79]. ACIP stated that the 9vHPV vaccine may be used to continue or complete a series started with a different HPV vaccine product, but no formal ACIP recommendations were released for subjects previously fully vaccinated with qHPV or bHPV vaccines who are willing to receive 9vHPV in order to benefit from broader protection. On the contrary, the European summary of product characteristics of the three HPV vaccines states that individuals who received a first dose with a given HPV vaccine should complete the vaccination course with that same vaccine [80, 81]. As we have described, one RCT has assessed 9vHPV vaccine for safety and immunogenicity in prior qHPV vaccine recipients and showed it to be well tolerated but to elicit an immune response against HPV types 31/33/45/52/58 lower than in girls who have never been exposed to HPV [36]. Experts recently combined available data with their opinion and judgment and concluded that – considering age at the start of vaccination, the number of doses already received and time interval between doses – 9vHPV might be used to complete an incomplete immunization regimen as well as added to a previous completed schedule to extend protection [80].

Last but not the least economic considerations should be mentioned. Several cost-effectiveness exercises projected potential health service savings derived by introduction of universal 9vHPV immunization programmes in different epidemiological settings and under different assumptions of infection transmission, vaccine efficacy, cross-protection, vaccine coverage, and costs. As emerges from different scenarios’ analysis, 9vHPV cost-effectiveness as compared with qHPV will depend on broader protection against HPV types but also on different duration of protection and cost per dose [28, 82–84].

Our study has both strengths and limitations. With regard to the latter, we could not carry out a quantitative pooling of retrieved findings (i.e. a meta-analysis) due to the heterogeneity of included studies. In the long run, when other studies – including of observational study design – become available, it will be useful to update the current study, possibly including implementation and effectiveness data. Since 9vHPV was licensed, a few narrative reviews and experts opinions have reported and commented on available efficacy, immunogenicity, and safety data, as well as on potential 9vHPV public health impact [85–87]. However, to the best of our knowledge, this has never been carried out in a systematic way. In this systematic review, we provide a comprehensive and critical update on the published available evidence as well as on the present status of ongoing research on relevant clinical and public health aspects of 9vHPV.

## CONCLUSION

The new 9vHPV vaccine appears to be non-inferior to other existing HPV vaccines in terms of safety and short-term immunogenicity and efficacy against common HPV types. The inclusion of additional HPV types in the vaccine offers great potential to expand protection against HPV infection and associated disease burden. However, 9vHPV impact in reducing the global burden of HPV-related cancer will greatly depend on vaccine uptake and coverage, availability, and – last but not least – affordability. For this to happen, international and national health authorities should engage in planning, implementing and evaluating effective immunization programmes, as well as invest in increasing the knowledge and awareness of HPV prevention among providers, parents, and people receiving the vaccine.

## Supplementary material

For supplementary material accompanying this paper visit http://dx.doi.org/10.1017/S0950268817000747. click here to view supplementary material
