# Supplementary material for: Human papillomavirus 9-valent vaccine for cancer prevention: a systematic review of the available evidence
Source: Epidemiol Infect. 2017 Apr 27;145(10):1962–82. doi: 10.1017/S0950268817000747 (PMC5974698; doi:10.1017/S0950268817000747)
Supplement: Supplementary file 1 [file S0950268817000747sup001.doc]

EPIDEMIOLOGY AND INFECTION

Human papillomavirus 9-valent vaccine for cancer prevention:

a systematic review of the available evidence

*A Systematic review on HPV-9 vaccine efficacy, immunogenicity and safety*

C. SIGNORELLI ^1,2^, A. ODONE ^1,3^, V. CIORBA ^1^, P. CELLA^1^, R.A. AUDISIO ^4^, A. LOMBARDI ^5^, L. MARIANI ^6^, F. S. MENNINI ^7.8^, S. PECORELLI ^9^, G. REZZA ^10^, G.V. ZUCCOTTI ^11^, A. PERACINO ^5^

Supplementary Material

**Supplementary Table S1.** Search strategies

**Medline Search strategy**

| **SET** | **Key words (free text)** | **Field** |
| --- | --- | --- |
| 1  2  3  4  5  6  7  8  9  10  11  12  13  14  15  16  17  18  19  20  21  22  23  24  25  26  27  28  29  30  31  32  33  34 | “Gardasil 9”  “Gardasil9”  “Gardasil-9”  v503  9vHPV  “9v HPV”  9v HPV vaccin*  nine valent HPV vaccin*  ninevalent HPV vaccin*  nine-valent HPV vaccin*  9 valent HPV vaccin*  9-valent HPV vaccin*  nonavalent HPV vaccin*  nine valent human papillomavirus vaccin*  ninevalent human papillomavirus vaccin*  nine-valent human papillomavirus vaccin*  9 valent human papillomavirus vaccin*  9-valent human papillomavirus vaccin*  nonavalent human papillomavirus vaccin*  human papillomavirus nine valent vaccin*  human papillomavirus ninevalent vaccin*  human papillomavirus nine-valent vaccin*  human papillomavirus 9 valent vaccin*  human papillomavirus 9-valent vaccin*  human papillomavirus nonavalent vaccin*  HPV 9 vaccin*  HPV9 vaccin*  HPV-9 vaccin*  HPV 9 valent vaccin*  HPV 9-valent vaccin*  HPV nine valent vaccin*  HPV ninevalent vaccin*  HPV nine-valent vaccin*  HPV nonavalent vaccin* | **[tile, abstract]** |
| 35 | **Sets 1-34 were combined with “OR”** |  |
| 36 | **Set 35 was limited to 25^th^ August 2016** |  |

**Embase Search strategy**

| **SET** | **Key words (free text)** | **Field** |
| --- | --- | --- |
| 1  2  3  4  5  6  7  8  9  10  11  12  13  14  15  16  17  18  19  20  21  22  23  24  25  26  27  28  29  30  31  32  33  34 | ‘Gardasil 9’  ‘Gardasil9’  ‘Gardasil-9’  v503  9vHPV  ‘9v HPV’  ‘9v HPV vaccin*’  ‘nine valent HPV vaccin*’  ‘ninevalent HPV vaccin*’  ‘nine-valent HPV vaccin*’  ‘9 valent HPV vaccin*’  ‘9-valent HPV vaccin*’  ‘nonavalent HPV vaccin*’  ‘nine valent human papillomavirus vaccin*’  ‘ninevalent human papillomavirus vaccin*’  ‘nine-valent human papillomavirus vaccin*’  ‘9 valent human papillomavirus vaccin*’  ‘9-valent human papillomavirus vaccin*’  ‘nonavalent human papillomavirus vaccin*’  ‘human papillomavirus nine valent vaccin*’  ‘human papillomavirus ninevalent vaccin*’  ‘human papillomavirus nine-valent vaccin*’  ‘human papillomavirus 9 valent vaccin*’  ‘human papillomavirus 9-valent vaccin*’  ‘human papillomavirus nonavalent vaccin*’  ‘HPV 9 vaccin*’  ‘HPV9 vaccin*’  ‘HPV-9 vaccin*’  ‘HPV 9 valent vaccin*’  ‘HPV 9-valent vaccin*’  ‘HPV nine valent vaccin*’  ‘HPV ninevalent vaccin*’  ‘HPV nine-valent vaccin*’  ‘HPV nonavalent vaccin*’ | **[tile, abstract]** |
| 35 | **Sets 1-34 were combined with ‘OR’** |  |
| 36 | **Set 35 was limited to 25^th^ August 2016** |  |

**The Cochrane Library search strategy**

| **SET** | **Key words (free text)** | **Field** |
| --- | --- | --- |
| 1  2  3  4  5  6  7  8  9  10  11  12  13  14  15  16  17  18  19  20  21  22  23  24  25  26  27  28  29  30  31  32  33  34 | “Gardasil 9”  “Gardasil9”  “Gardasil-9”  v503  9vHPV  “9v HPV”  “9v HPV vaccine”  “nine valent HPV vaccine”  “ninevalent HPV vaccine”  “nine-valent HPV vaccine”  “9 valent HPV vaccine”  “9-valent HPV vaccine”  “nonavalent HPV vaccine”  “nine valent human papillomavirus vaccine”  “ninevalent human papillomavirus vaccine”  “nine-valent human papillomavirus vaccine”  “9 valent human papillomavirus vaccine”  “9-valent human papillomavirus vaccine”  “nonavalent human papillomavirus vaccine”  “human papillomavirus nine valent vaccine”  “human papillomavirus ninevalent vaccine”  “human papillomavirus nine-valent vaccine”  “human papillomavirus 9 valent vaccine”  “human papillomavirus 9-valent vaccine”  “human papillomavirus nonavalent vaccine”  “HPV 9 vaccine”  “HPV9 vaccine”  “HPV-9 vaccine”  “HPV 9 valent vaccine”  “HPV 9-valent vaccine”  “HPV nine valent vaccine”  “HPV ninevalent vaccine”  “HPV nine-valent vaccine”  “HPV nonavalent vaccine” | [Title, abstract] |
| 45 | **Sets 1-34 were combined with “OR”** |  |
| 46 | **Set 35 was limited to 25^th^ August 2016** |  |

**ClinicalTrials.gov search strategy**

| **SET** | **Key words (free text)** | **Field** |
| --- | --- | --- |
| 1  2  3  4  5  6  7 | “Gardasil 9”  “Gardasil9”  “Gardasil-9”  “v503”  “9vHPV”  “9v HPV”  “9v HPV vaccin*” | **[search terms]** |
| 8 | **Sets 1-7 were combined with “OR”** |  |
| 9 | **Set 8 was limited to 25^th^ August 2016** |  |

**International Clinical Trials Registry Platform search strategy**

| **SET** | **Key words (free text)** | **Field** |
| --- | --- | --- |
| 1  2  3  4  5  6  7  8  9  10  11  12  13  14  15  16  17  18  19  20  21  22  23  24  25  26  27  28  29  30  31  32  33  34 | “Gardasil 9”  “Gardasil9”  “Gardasil-9”  v503  9vHPV  “9v HPV”  9v HPV vaccin*  nine valent HPV vaccin*  ninevalent HPV vaccin*  nine-valent HPV vaccin*  9 valent HPV vaccin*  9-valent HPV vaccin*  nonavalent HPV vaccin*  nine valent human papillomavirus vaccin*  ninevalent human papillomavirus vaccin*  nine-valent human papillomavirus vaccin*  9 valent human papillomavirus vaccin*  9-valent human papillomavirus vaccin*  nonavalent human papillomavirus vaccin*  human papillomavirus nine valent vaccin*  human papillomavirus ninevalent vaccin*  human papillomavirus nine-valent vaccin*  human papillomavirus 9 valent vaccin*  human papillomavirus 9-valent vaccin*  human papillomavirus nonavalent vaccin*  HPV 9 vaccin*  HPV9 vaccin*  HPV-9 vaccin*  HPV 9 valent vaccin*  HPV 9-valent vaccin*  HPV nine valent vaccin*  HPV ninevalent vaccin*  HPV nine-valent vaccin*  HPV nonavalent vaccin* | **[title]** |
| 35 | **Sets 1-34 were combined with “OR”** |  |
| 36 | **Set 35 was limited to 25^th^ August 2016** |  |

**EU Clinical Trials Register search strategy**

| **SET** | **Key words (free text)** | **Field** |
| --- | --- | --- |
| 1  2  3  4  5  6  7 | “Gardasil 9”  “Gardasil9”  “Gardasil-9”  “v503”  “9vHPV”  “9v HPV”  “9v HPV vaccin*” | **[search terms]** |
| 8 | **Sets 1-7 were combined with “OR”** |  |
| 9 | **Set 8 was limited to 25^th^ August 2016** |  |
